# TARDBP is a candidate diagnostic biomarker promoting tumor progression via impacting tumor immunity and tumor microenvironment

**DOI:** 10.7150/jca.96800

**Published:** 2024-06-03

**Authors:** Min-Yi Luo, Zhe Han, Jiaqi Wang, Cheng Zhong, Jiancong Chen

**Affiliations:** 1 Department of Pancreato-Biliary Surgery, The First Affiliated Hospital, Sun Yat-sen University, 510080, Guangzhou, Guangdong, China.; 2 Department of Coloproctology, The Sixth Affiliated Hospital, Sun Yat-sen University, 510080, Guangzhou, Guangdong, China.; 3 Department of Neurology, The First Bethune Hospital of Jilin University, 130000, Changchun, Jilin, China.; 4 Department of Neonatology, Jiangmen People's Hospital, 52900, Jiangmen, Guangdong, China.; 5 Department of Orthopedics, Jiangmen Hospital of Traditional Chinese Medicine Affiliated to Jinan University, 52900, Jiangmen, Guangdong, China.

## Abstract

In the realm of cancer research, particularly hepatocellular carcinoma (HCC), TAR DNA-binding protein (TARDBP) has transitioned from being associated with neurodegenerative diseases to emerging as a significant molecule in oncology due to its aberrant expression in HCC and other malignancies. This shift underlines the versatility of TARDBP and its critical role in tumorigenesis. Our study illuminates TARDBP's universal upregulation across various cancers, indicating its involvement in fundamental oncogenic processes and potential impact on genomic instability. The relationship between TARDBP expression and tumor mutational burden (TMB) across several cancers highlights its influence on a key hallmark of cancer progression. Additionally, TARDBP's interaction with immune and inflammatory factors within the tumor microenvironment, including its association with immune-stimulatory factors and inverse relationship with immune inhibitors, suggests its role in modulating immune evasion. Clinically, TARDBP's aberrant expression correlates with adverse patient outcomes in HCC, making it a promising candidate for therapeutic targeting. The study concludes that TARDBP holds significant potential as a novel therapeutic target in HCC and possibly other malignancies, meriting further exploration to integrate TARDBP-targeted therapies into cancer treatment protocols, thereby advancing the field of precision medicine.

## 1. Introduction

TAR DNA-binding protein 43 (TARDBP), also known as TDP-43, is a highly conserved nuclear protein involved in the regulation of gene expression, including roles in RNA splicing, transcriptional repression, and RNA stability 1, 2. Beyond its well-documented involvement in neurodegenerative diseases, emerging evidence has highlighted the significance of TARDBP in various cancer pathways, indicating its potential as a biomarker for tumor progression and prognosis 3-7. The protein's aberrant expression and mutations have been associated with altered cellular dynamics, promoting oncogenesis through the dysregulation of cell proliferation, apoptosis, and metastasis 8-10. Studies have demonstrated that TARDBP can influence tumor microenvironment, angiogenesis, and immune escape mechanisms, thereby supporting the aggressive behavior of malignant cells 11, 12. Despite its growing recognition in cancer biology, the comprehensive impact of TARDBP across different cancer types, particularly in solid tumors, remains inadequately explored.

Hepatocellular carcinoma (HCC) stands as a primary malignancy of the liver, representing one of the most common and aggressive forms of liver cancer. The pathogenesis of HCC is a complex process involving multiple genetic and epigenetic alterations, chronic inflammation, and exposure to environmental risk factors 13-15. Current therapeutic strategies for HCC are limited by the late diagnosis in most patients and the high rate of tumor recurrence and metastasis 16, 17. The urgent need for novel diagnostic markers and therapeutic targets has propelled extensive research into the molecular underpinnings of HCC. In this context, the role of key regulatory proteins, such as TARDBP, has come under scrutiny 16, 18. Preliminary studies suggest a potential link between TARDBP expression levels and HCC progression, but the underlying mechanisms and the protein's prognostic value in HCC remain to be fully elucidated.

TARDBP's involvement in cancer appears to be multifaceted, influencing various hallmarks of cancer across different tumor types. In neurodegenerative conditions, TARDBP has been implicated in the regulation of neuronal cell death, highlighting its potential role in cell survival pathways that are often hijacked in cancer 10, 19, 20. This pan-cancer analysis of TARDBP seeks to delineate its expression patterns, mutational landscape, and functional impacts across a broad spectrum of cancers, with a particular focus on HCC. By integrating high-throughput genomic and transcriptomic data, this study aims to uncover the molecular mechanisms by which TARDBP contributes to tumorigenesis and tumor progression. Furthermore, the research will evaluate TARDBP's utility as a prognostic marker for HCC, potentially offering new insights into the management of this challenging disease. Ultimately, this study is dedicated to exploring the role of TARDBP in cancer biology, bridging the gaps in the current literature, and providing new insights for future therapeutic interventions.

## 2. Materials and Methods

### 2.1. Data collection and processing

The genomic profiles, clinicopathological characteristics, and somatic mutation datasets, along with tumor mutational burden (TMB) and microsatellite instability (MSI) metrics for thirty-three pan-cancer types from The Cancer Genome Atlas (TCGA) Genomic Data Commons (GDC), were acquired from the University of California, Santa Cruz (UCSC) Xena platform (https://xenabrowser.net/ accessed on 10.02.2024). Prior to analysis, normalization of expression data was conducted using the Fragment Per Kilobase Million (FPKM) method. Tumor mutational burden was quantified by enumerating somatic mutations per million base pairs within non-repetitive genomic regions. Microsatellite instability was assessed by calculating the frequency of length polymorphisms due to insertion or deletion mutations per million base pairs.

### 2.2. Gene expression in pan cancer between cancers and normal tissues

A comparative analysis of TARDBP mRNA levels in malignant versus non-malignant tissues was conducted utilizing the R packages “limma” for differential expression analysis and “TCGAplot” for data visualization across multiple cancer subtypes. Data pertaining to tumor mutational burden (TMB) and microsatellite instability (MSI) were sourced from The Cancer Genome Atlas (TCGA) repository. We applied Spearman's rank correlation coefficient to explore potential correlations between TARDBP gene expression and both TMB and MSI indices. All statistical analyses were performed in the R programming environment. The outcomes were graphically represented through radar charts, facilitated by the “fmsb” package in R, to provide a multidimensional view of the expression patterns relative to TMB and MSI across different tumor contexts.

### 2.3. Immunorelational analysis of TARDBP and correlation with tumor microenvironment

Utilizing the R packages “reshape2” for data restructuring and “RColorBrewer” for visual enhancement, we analyzed the association between TARDBP gene expression and immune checkpoint genes. The correlation between TARDBP expression and various immune regulatory components, including checkpoints, chemokines, chemokine receptors, immustimulators, immuinhibitors, and immucells, was explored using Spearman's rank correlation test. The significant associations were visualized as heatmaps, with a p-value threshold of less than 0.05 considered statistically significant.

Further, the ESTIMATE (Estimation of Stromal and Immune cells in Malignant Tumor tissues using Expression data) scoring system was employed to evaluate the presence of stromal and immune cells in tumor tissues. This sophisticated tool computed ImmuneScores, StromalScores, and ESTIMATEScores for thirty-three different cancer types cataloged in The Cancer Genome Atlas (TCGA). These scores reflect the respective proportions of stromal and immune components within the tumor microenvironment (TME), with higher scores indicating a greater presence of the respective cell type. The ESTIMATEScore, representing the combined proportion of stromal and immune cells, was calculated as the sum of the ImmuneScore and StromalScore.

Using the "estimate" R package, ImmuneScores and StromalScores, along with tumor purity, were estimated. Visualization of the data involved the "ggplot2", "ggpubr", "ComplexHeatmap", and "ggExtra" packages to construct comprehensive visual representations, including triangular heatmaps. Data preprocessing steps such as normalization and filtering were conducted to ensure reliable analyses. Finally, correlation analyses using Spearman's test further assessed the relationship between TARDBP expression and both ImmuneScores and StromalScores, highlighting their potential roles in the TME dynamics.

### 2.4. Clinical relevance analysis of TARDBP

Univariate Cox regression analysis was conducted on TCGA datasets using the R packages “survival” and “forestplot” to evaluate the prognosis value of TARDBP regarding aspects of survival time. The investigation utilized data obtained from The Cancer Genome Atlas (TCGA). R statistical software environment and the ggplot2 package were employed to plot boxplots illustrating the differential expression of TARDBP in LIHC compared to normal populations and the differential expression of TARDBP in LIHC patients across different genders. And we obtained paired boxplots illustrating the differential expression of TARDBP in matched tumor and normal tissues of LIHC using the "ggpubr" package. We utilized the "ggpubr" package to generate visual boxplots and conducted statistical tests using the "stat_compare_means" function to obtain boxplots depicting the differential expression of TARDBP in LIHC patients across different age groups and WHO stages. Then, plot the ROC curve of TARDBP in LIHC.

Univariate Cox regression analysis, utilizing the "survival" and "forestplot" R packages, was conducted to assess the prognostic relevance of TARDBP expression based on survival data from The Cancer Genome Atlas (TCGA). This analysis aimed to elucidate the potential of TARDBP as a prognostic biomarker in various cancers.

Additionally, the differential expression of TARDBP in liver hepatocellular carcinoma (LIHC) relative to normal liver tissues was investigated using the "limma" and "ggplot2" packages. Boxplots, crafted to clearly depict these differences, were generated for visual analysis. Comparisons of matched tumor and normal tissues in LIHC patients were illustrated using paired boxplots created with the "ggpubr" package. This package also enabled the visualization of TARDBP expression variations across different genders, age groups, and World Health Organization (WHO) cancer stages within the LIHC cohort.

Moreover, the diagnostic potential of TARDBP in LIHC was explored by plotting a Receiver Operating Characteristic (ROC) curve. This analysis provided valuable insights into the efficacy of TARDBP as a diagnostic biomarker. The comprehensive methodology highlights the extensive utility of TARDBP analysis in both the prognosis and diagnosis of cancer, underscoring its significance in oncological research.

### 2.5. Co-expression analysis and GSEA (Gene Set Enrichment Analysis) of TARDBP in LIHC patients

LIHC patients were divided into low- and high-expression groups based on TARDBP median expression levels. We performed differential analysis using the "limma" and “TCGAplot” package, followed by heatmap generation to identify co-expressed genes positively and negatively correlated with TARDBP in LIHC patients. Subsequently, we utilized the "ggplot2" package to conduct Gene Ontology (GO) enrichment analysis separately for the co-expressed genes positively and negatively correlated with TARDBP in LIHC patients, resulting in two bubble plots. R package “ClusterProfiler” and “GSVA” packages were used to perform the Enrichment Analysis. Results were then visualized using the R package “Corrgram”.

### 2.6. Cell culture

Human hepatocellular carcinoma cells (Huh7) were kindly provided by the Shanghai Institutes for Biological Sciences, Chinese Academy of Sciences (ATCC, Shanghai, China). Additionally, human hepatoma cells (HepG2) were obtained from the cell bank of the Chinese Academy of Sciences (Shanghai, China). The Huh7 and HepG2 cell lines were propagated in DMEM media (Gibco, Thermo Fisher Scientific, Waltham, MA, USA) enriched with 10% fetal bovine serum (ABW, AB-FBS-1050S) and 1% penicillin-streptomycin (Invitrogen, Carlsbad, CA, USA). Cultivation conditions were maintained at 37 °C in an atmosphere of 5% CO2 and 80% humidity. Only cells in the logarithmic phase of growth nearing 80% confluence were utilized for subsequent experimental procedures.

### 2.7. RNA isolation and quantitative Reverse Transcription-Polymerase Chain Reaction (qRT-PCR) analysis

Quantitative real-time PCR (qRT-PCR) confirmed the findings post RNA extraction from human hepatocellular carcinoma cells (Huh7) and human hepatoma cells (HepG2), along with TARDBP-transfected Huh7 or HepG2 cells. Primer sequences for the targeted genes, listed in Supplementary Table S2, were synthesized by RiboBio (Guangzhou, China). Each primer's specific melting and annealing temperatures are detailed in Supplementary Table S2. The EZ-press RNA Purification Kit (EZBioscience, ZScience Biotechnology Corporation Limited, Roseville, MN, USA) facilitated total RNA extraction. A NanoDrop 2000 spectrometer evaluated the RNA's purity and concentration. The RT-qPCR reactions utilized the 4×Reverse Transcription Master Mix (EZBioscience, China) and 2×SYBR Green qPCR Master Mix (EZBioscience, Guangzhou, China), following manufacturer protocols on the Applied Biosystems™ QuantStudio™ 5 Real-Time PCR System. Graphpad Prism 9.0 software aided in figure illustration. GAPDH served as the internal control, employing the 2-ΔΔCt method for normalizing targeted gene expression, which was strategically chosen during the logarithmic growth phase at approximately 80% confluence.

### 2.8. Western blotting analysis

RIPA lysis buffer (Beyotime, Shanghai, China) with a Protease Inhibitor Cocktail (CoWin Biosciences, Jiangsu, China) (1:100) lysed both HepG2 and Huh7 cell lines. The total protein content was quantified using a bicinchoninic acid (BCA) protein assay kit (Thermo Scientific). Subsequently, 10 μg of each protein sample underwent separation via 10% sodium dodecyl sulfate-polyacrylamide gel electrophoresis (SDS-PAGE). Total protein transfer post-electrophoresis was facilitated by a 0.45 μm polyvinylidene difluoride membrane. Membranes underwent blocking in a protein-free rapid blocking buffer (Epizyme Biotech, PS108) for 10-15 minutes. Primary and secondary antibodies were sequentially incubated, the former overnight at 4 °C and the latter for 1 hour at room temperature, before an ECL chromogenic kit (Thermo Fisher Scientific) detected the antibody binding. Chemiluminescence and the Fusion FX5 image analyzer (Vilber Lourmat, Marne La Vallée, France) visualized the antibody binding. The primary antibodies used were TARDBP (Proteintech, Wuhan, China), GAPDH (CST, New York, NY, USA), and β-Actin (CST).

### 2.9. siRNA interference assay and CCK-8 assay

HanYi Biosciences Inc., China, designed two TARDBP-siRNAs (Supplementary Table S3). Transfections of HepG2 or Huh7 with siRNAs followed the jetPRIME (Polyplus, Illkirch, French) manufacturer's instructions. A functional assay ensued 48 hours post-transfection to collect proteins and RNA. Cell proliferation was evaluated using the Cell Counting Kit-8 (Dojindo, Japan, CK04-5) following transfection with TARDBP-siRNA. Cells were seeded in a 96-well plate, and a microplate reader (Bio-Rad Laboratories, Hercules, CA, USA) measured cell viability at intervals of 1, 2, 3, 4, and 5 days.

### 2.10. Wound healing, cell migration assays, and colony formation test

A pipette tip created a scratch in the cell monolayer within a six-well plate. Images were captured at 0 and 76 hours post-scratching. To assess the migration ability of SNU449 or Huh7, cells were starved in serum-free RPMI 1640 medium for 8 hours. Subsequently, 5 × 10^4 cells in 100 μL of serum-free RPMI 1640 medium were introduced into transwell inserts (Corning, Corning, NY, USA). Serum-free RPMI 1640 medium supplemented with 10% FBS served as a chemoattractant for the transwell assays. After 8 hours, cells on the lower surface were fixed with 4% paraformaldehyde (Beyotime) for 30 minutes and stained with 0.4% crystal violet (Beyotime). The upper surface was cleaned with a cotton swab, and cells on the lower surface were counted under a microscope. SNU449 or Huh7 cells transfected with TARDBP-siRNA were incubated in RPMI-1640 medium supplemented with 10% FBS at 37 °C in a 5% CO2 atmosphere. Within 24 hours of transfection, 1,000 cells were plated into six-well plates and cultured in RPMI-1640 medium with 10% FBS for two weeks before colonies were counted and analyzed.

### 2.11. Statistical analysis

The Kruskal-Wallis test and Wilcoxon rank-sum test assessed differences in gene expression levels between tumors and normal tissues. Two-tailed tests set the significance threshold at p < 0.05 for all parametric analyses in this study. R software (version 4.1.3, R Foundation for Statistical Computing), available through the CRAN mirror hosted by Tsinghua University, Beijing, China (https://www.r-project.org/), performed statistical analyses as of March 4, 2023. The Fisher's exact test and the t-test, assuming equal variances, evaluated group comparisons for categorical and continuous variables, unless otherwise noted. We examined the diagnostic accuracy of gene expression levels in predicting preeclampsia through ROC curve analysis, calculating AUC values. Statistical significance was determined at p < 0.05, unless specified differently.

## Results

### 3.1. Landscape of TARDBP expression in pan-cancer and normal tissues

**Figure 1:**
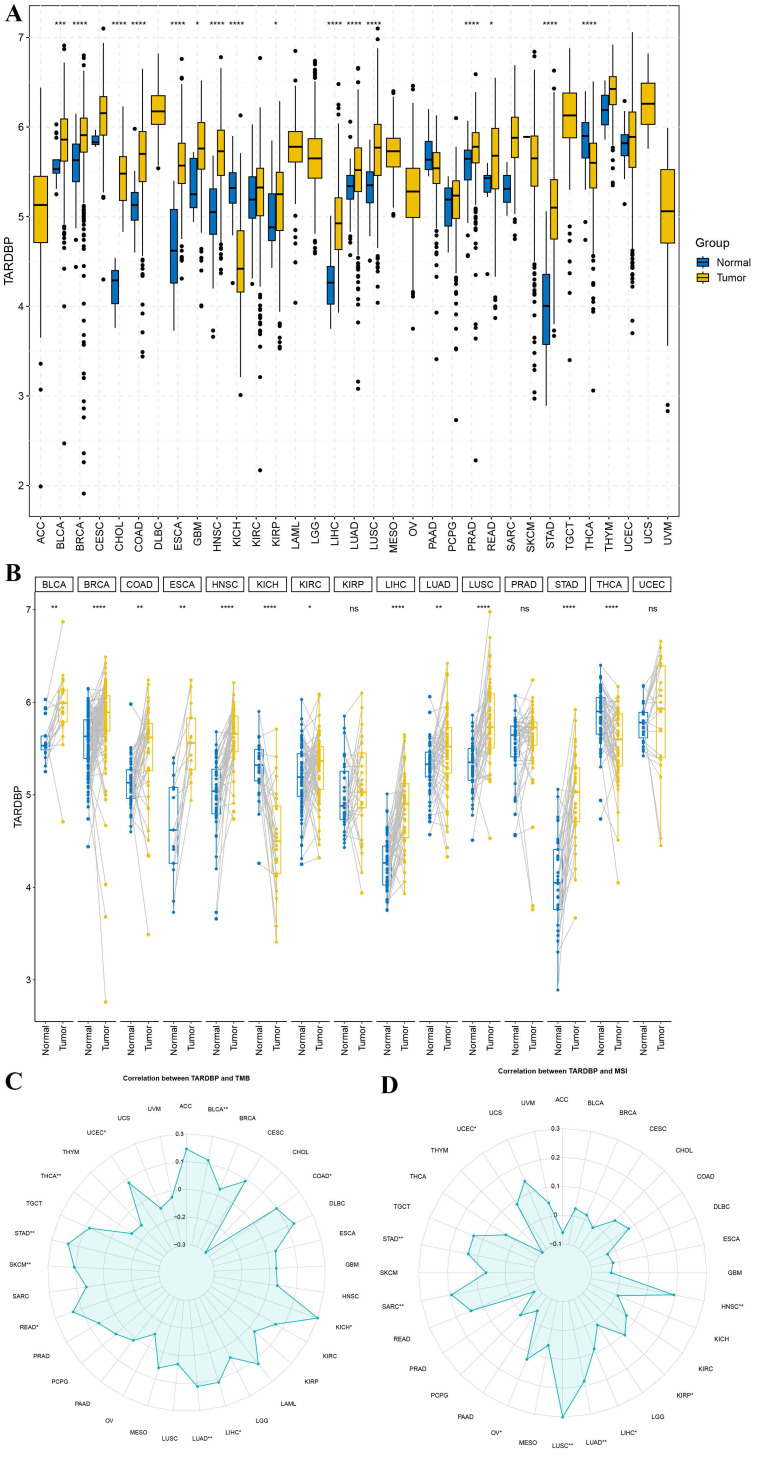
(A) Expression levels of TARDBP in pan-cancers were analyzed in the TCGA database. (B) Expression of TARDBP in paired samples of 33 tumors in the TCGA database. (C, D) Relationships between TARDBP gene expression and TMB (D), MSI (E) in different types of cancers.

We conducted an analysis of TARDBP gene distribution and expression across various tumor tissues. Despite the absence or limited availability of normal tissue controls in several cancer types, significant differential expression of TARDBP was observed in 16 of the 33 cancers analyzed, including notable examples such as BRCA, CHOL, COAD, ESCA, HNSC, LUAD, LUSC, PRAD, STAD, and THCA, as depicted in Figure 1A.

Further investigations into TARDBP expression were carried out in matched cancerous and adjacent normal tissues. This analysis revealed an upregulation of TARDBP in 12 out of the 15 cancer types examined. Conversely, pairwise comparisons for specific cancers including BRCA, HNSC, KICH, LIHC, LUSC, STAD, and THCA did not yield statistically significant results (Figure 1B).

To explore the relationship between TARDBP expression and tumor mutational burden (TMB) as well as microsatellite instability (MSI), a correlation analysis was performed. TARDBP expression was positively correlated with TMB in BLCA, KICH, LIHC, LUAD, READ, SKCM, STAD, and UCEC and negatively correlated with TMB in COAD and THCA (p < 0.05) (Figure 1C). Regarding MSI, a positive correlation was observed with TARDBP expression in cancers such as UCEC, STAD, SARC, OV, LUSC, LUAD, LIHC, KIRP, and HNSC (p < 0.05) (Figure 1D). These findings underscore the varied role of TARDBP in oncogenesis and its potential utility as a biomarker in cancer diagnosis and prognosis.

### 3.2. The correlation between TARDBP expression and immunological factors

**Figure 2:**
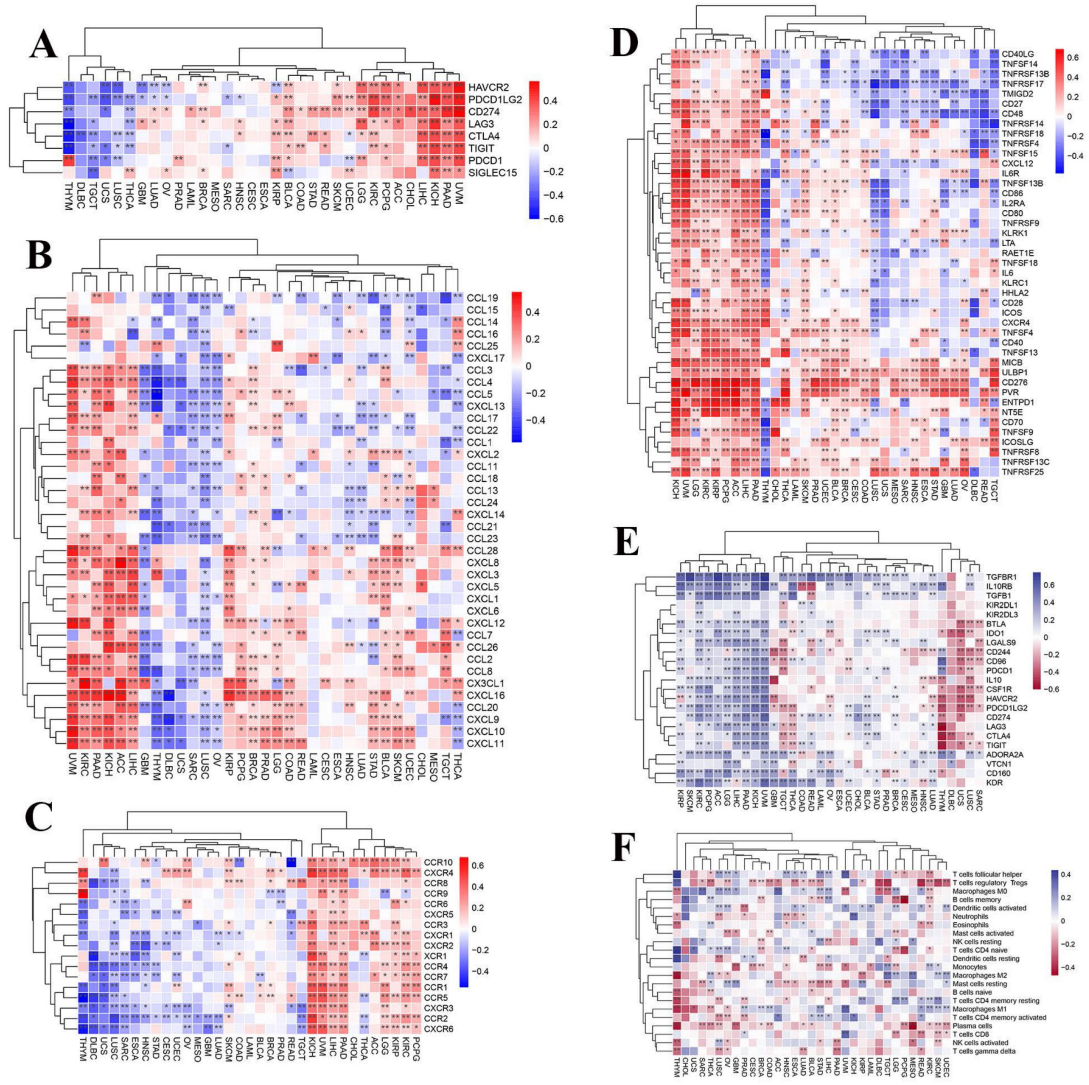
(A-F) Heatmap displaying correlations between TARDBP expression in 33 tumors and checkpoints(A), chemokines(B), chemokine receptors(C), immustimulators (D), immuinhibitors (E) and immucells (F).

To further investigate the presumable causes of aberrant TARDBP expression, we conducted an extensive analysis to investigate the relationship between immune and inflammatory-related factors across different cancer types. Following the analysis, we observed correlations between TARDBP expression and checkpoints (**Figure 2A**), chemokines (**Figure 2B**), receptors (**Figure 2C**), immustimulators (**Figure 2D**), immuinhibitors (**Figure 2E**) and immucells (**Figure 2F**) in various human cancers. TARDBP expression exhibited a positive association with checkpoints, chemokines, chemokine receptors and immustimulators while showing an inverse correlation with immuinhibitors. Based on the results, it is evident that TARDBP may play a crucial role in modulating the immune response within TME by influencing the behavior of Immunoregulatory pathway.

### 3.3. Correlation of TARDBP expression with tumor immune microenvironment

**Figure 3:**
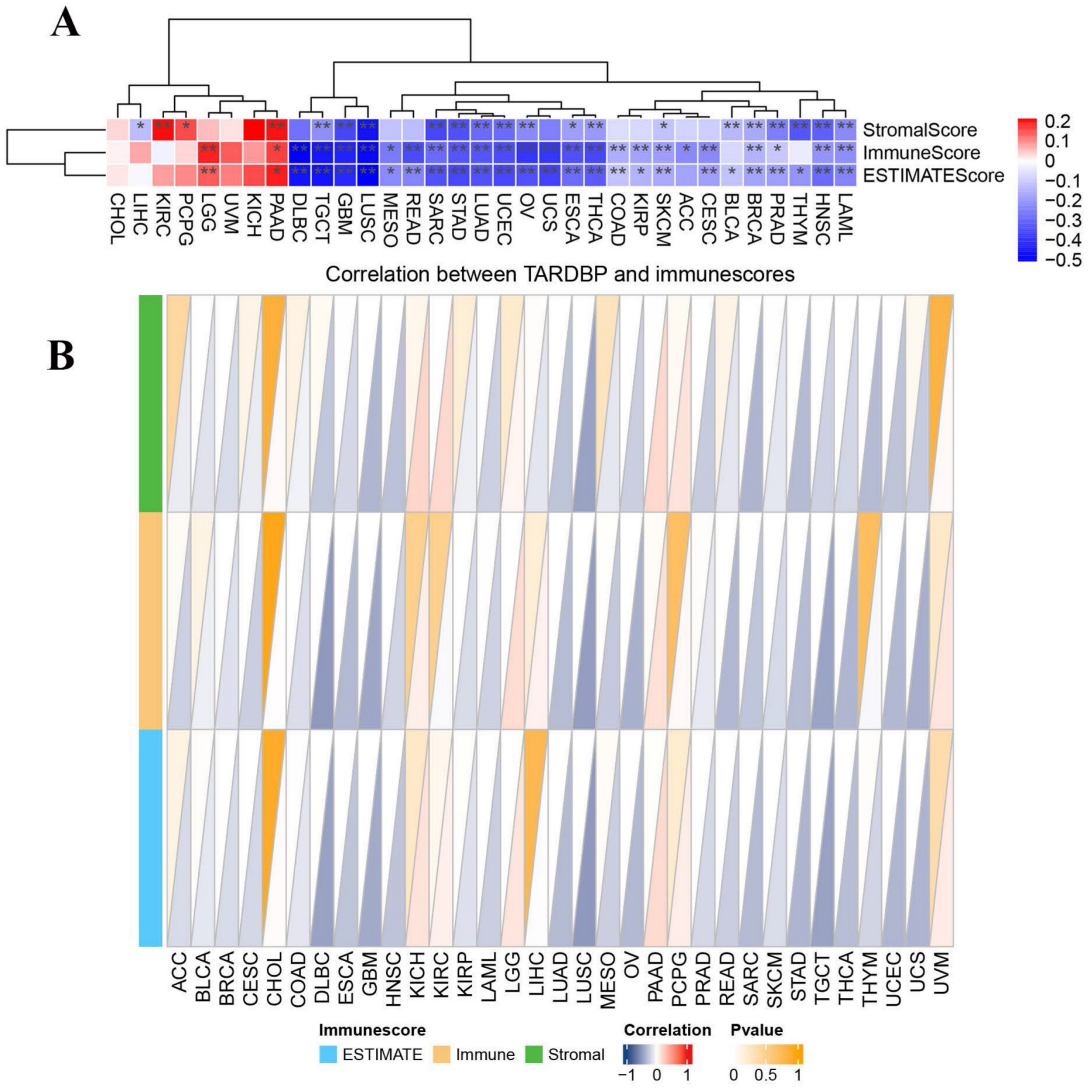
(A) Heatmap displaying correlations between TARDBP expression and immune, stromal, and ESTIMATE scores in pan-cancer; red represents positive correlation, and blue represents negative correlation. (B) Triangle Heatmap displaying correlation between TARDBP and immunescores.

It is critically important to further explore the pan-cancer relationship between TARDBP expression and the tumor microenvironment (TME). Employing the ESTIMATE algorithm, we calculated the StromalScore, ImmuneScore, and ESTIMATEScore for TARDBP across 33 cancer types. We observed a significant negative correlation between TARDBP expression and the StromalScore, ImmuneScore, and ESTIMATEScore in 14 different cancer types, specifically TGCT, GBM, LUSC, SARC, STAD, LUAD, UCEC, OV, ESCA, THCA, SKCM, BRCA, PRAD, HNSC, and LAML. Conversely, an opposite trend was noted in PAAD (Figure 3A).

Subsequently, we constructed a triangular heatmap to illustrate the correlation between TARDBP expression and immune scores. Notably, TARDBP expression was negatively correlated with immune scores in 17 different cancer types, including BRCA, DLBC, ESCA, GBM, HNSC, LAML, LUAD, LUSC, OV, PRAD, SARC, SKCM, STAD, TGCT, THCA, UCEC, and UCS. This observation underscores TARDBP's potential involvement in modulating immune responses within these cancerous contexts (Figure 3B), suggesting its role as an influential factor in the TME dynamics.

### 3.4. Analysis of the clinical relevance of TARDBP expression

**Figure 4:**
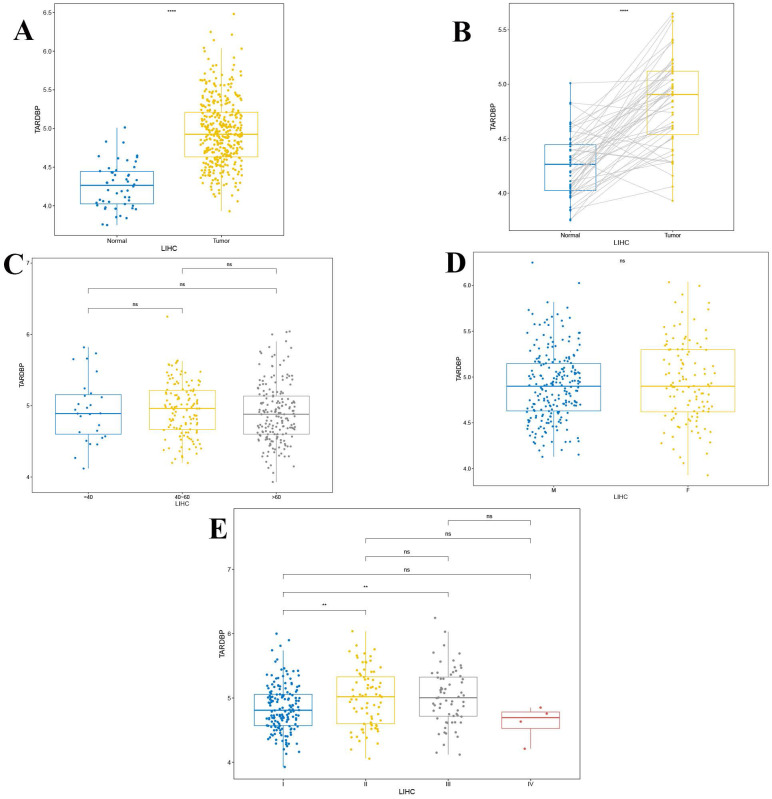
(A) The differential expression of TARDBP in LIHC compared to the normal population. (B) The differential expression of TARDBP in paired tumor and normal tissues of LIHC (Liver Hepatocellular Carcinoma). (C) The differential expression of TARDBP in patients with LIHC (Liver Hepatocellular Carcinoma) across different age groups. (D)The differential expression of TARDBP in patients with LIHC (Liver Hepatocellular Carcinoma) across different genders. (E) The differential expression of TARDBP in patients with LIHC (Liver Hepatocellular Carcinoma) across different WHO stages.

These findings demonstrate a significant upregulation of TARDBP in liver hepatocellular carcinoma (LIHC) compared to normal liver tissues, with pronounced differences evident in paired tumor versus normal tissue analyses (p < 0.0001) as depicted in Figures 4A and 4B. Additionally, no significant differences in TARDBP expression were observed across different age groups and genders within the LIHC cohort (Figures 4C and 4D). However, differential expression of TARDBP was notable between WHO stages I and II, as well as between stages I and III in LIHC patients (p < 0.01) as shown in Figure 4E.

**Figure 5:**
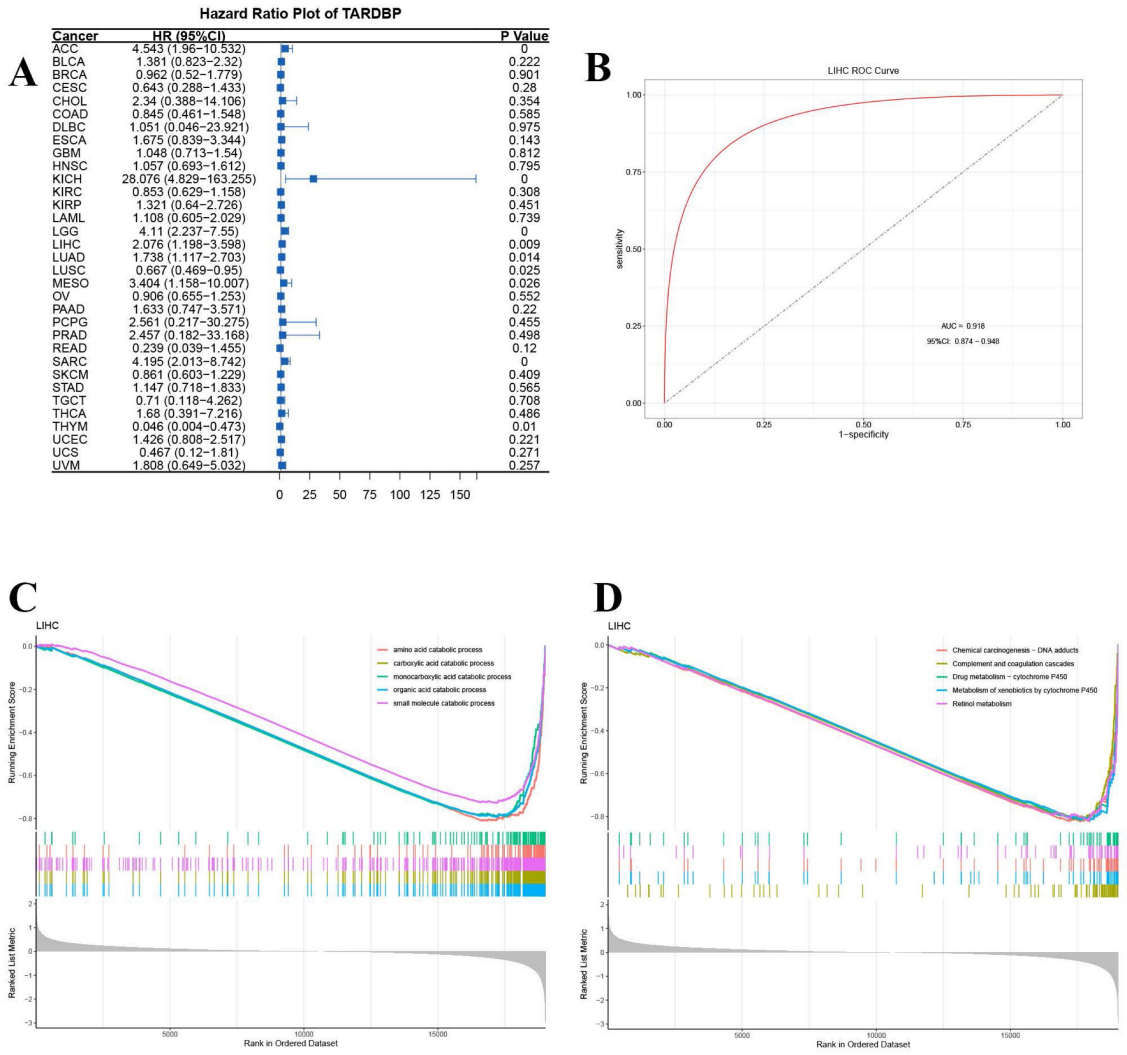
(A) Hazard Ratio Plot of TARDBP for survival time across different tumors. (B) ROC curve of TARDBP in LIHC. (C,D) GSEA for TARDBP. Different color curves represent different functions or pathways. The peak of the upward and downward curve indicates the positive and negative regulation of TARDBP.

Additionally, univariate Cox regression analysis was conducted to assess the impact of TARDBP expression on survival across multiple cancer types. The analysis revealed that the hazard ratios for TARDBP were significant in cancers such as adrenocortical carcinoma (ACC), kidney chromophobe (KICH), lower grade glioma (LGG), LIHC, lung adenocarcinoma (LUAD), mesothelioma (MESO), sarcoma (SARC), lung squamous cell carcinoma (LUSC), and thymoma (THYM), with KICH showing the most pronounced risk associated with increased TARDBP expression (Figure 5A). Furthermore, the area under the curve (AUC) of the ROC curve for TARDBP in LIHC was 0.918, indicating high diagnostic accuracy for predicting LIHC incidence based on TARDBP expression levels (Figure 5B).

GSEA Go analysis revealed significant downregulation of small molecule catabolic processing pathways across various cancers (Figure 5C). Additionally, GSEA-KEGG pathway enrichment analysis in LIHC demonstrated significant associations with pathways, such as chemical carcinogenesis-DNA adducts, complement and coagulation cascades, drug metabolism-cytochrome P450, metabolism of xenobiotics by cytochrome P450 and retinol metabolism (Figure 5D).

### 3.5. Enrichment of TARDBP co-expressed genes in various pathways

**Figure 6:**
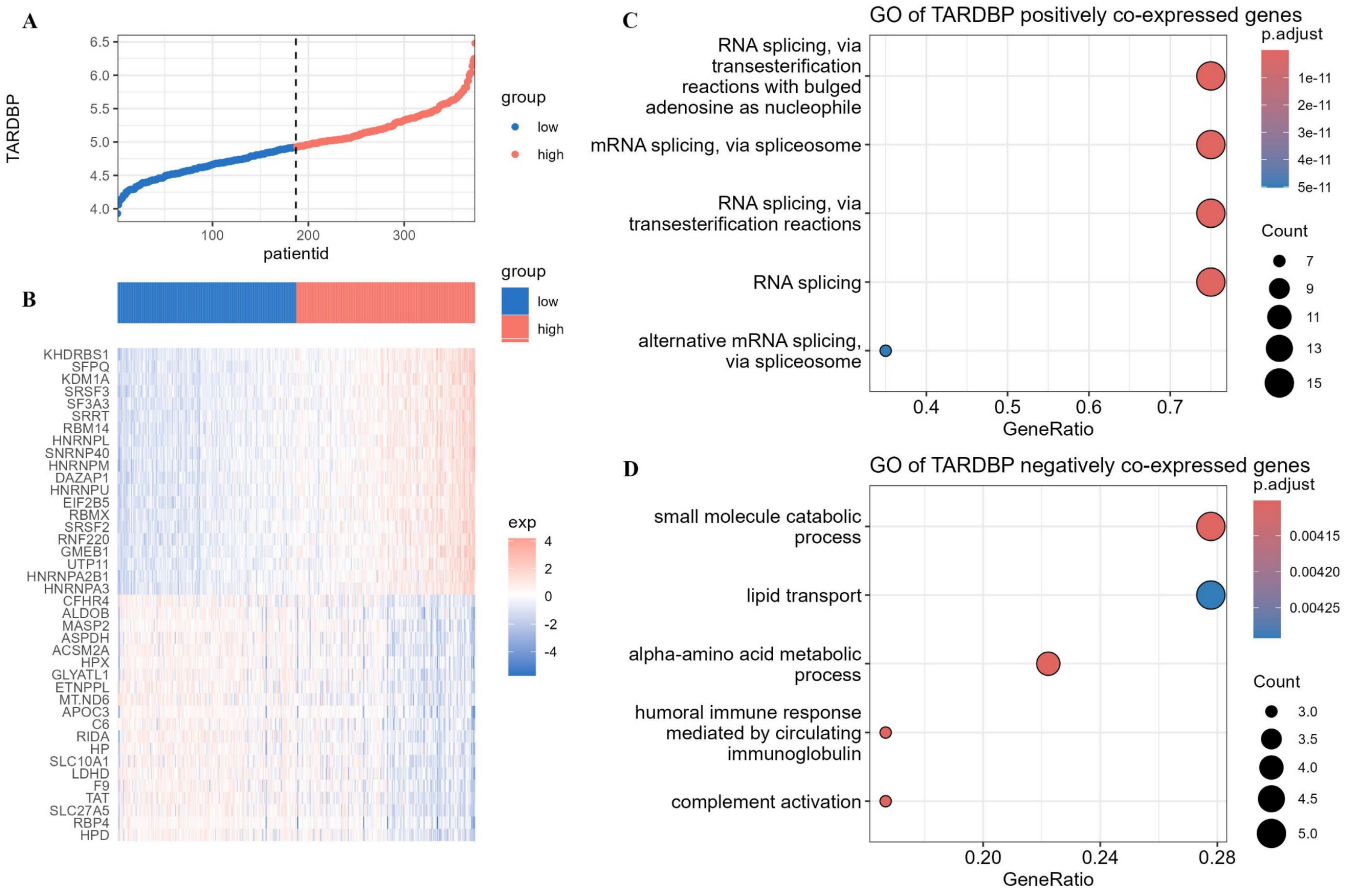
(A)The expression levels of TARDBP in different LIHC patients. (B)Heatmap depicting the differential expression of genes related to biological regulation in patients with high and low expression of the TARDBP gene. (C, D) Bubble plots depicting GO enrichment analysis of co-expressed genes positively and negatively correlated with TARDBP.

Using the median expression level of TARDBP in different LIHC patients as a standard, the LIHC population was divided into TARDBP high-expression group and TARDBP low-expression group (**Figure 6A**). We conducted differential analysis of the expression of genes related to biological regulation in patients with high and low expression of the TARDBP gene. We selected the top 20 genes from both positively and negatively correlated genes and generated heatmaps (**Figure 6B**). Subsequently, we performed GO enrichment analysis on the co-expressed genes positively and negatively correlated with TARDBP, respectively. The results showed that, in LIHC patients, TARDBP positively correlated co-expressed genes were most significantly enriched in the mRNA processing pathway (**Figure 6C**), while TARDBP negatively correlated co-expressed genes were most significantly enriched in the small molecule catabolic process and lipid transport pathway (**Figure 6D**).

### 3.6. Correlation between TARDBP expression and CD274 and CTLA4

**Figure 7:**
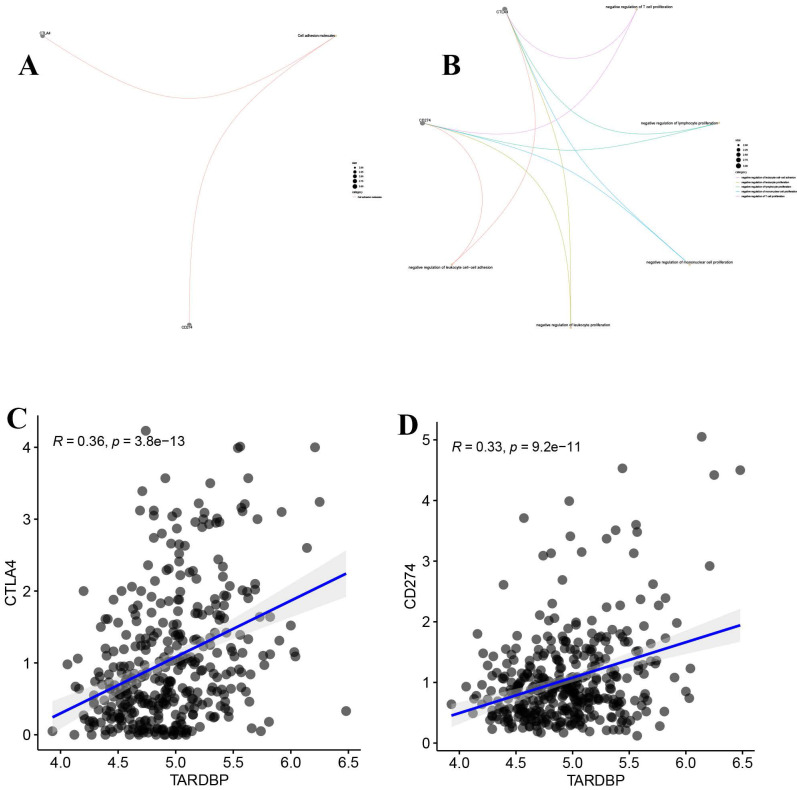
(A) The molecular properties of CD274 and CTLA4. (B) The functions of CD274 and CTLA4. (C) Scatter plot depicting the correlation between TARDBP and CTLA4 in LIHC. (D) Scatter plot depicting the correlation between TARDBP and CD274 in LIHC.

CD274 (PD-L1) and CTLA4 are immune checkpoint proteins that inhibit T cell activation, essential for controlling immune responses. Our analysis, illustrated in Figure 7A, shows their expression across various samples. Additionally, Figure 7B indicates their involvement in suppressing lymphocyte and leukocyte proliferation. Notably, Figures 7C and 7D reveal a significant positive correlation between TARDBP expression and both CD274 and CTLA4, suggesting a linkage in their regulatory effects within the tumor microenvironment.

### 3.7. TARDBP knockdown efficiency and its impact on mRNA and protein expression in HepG2 and Huh7 Cells

**Figure 8:**
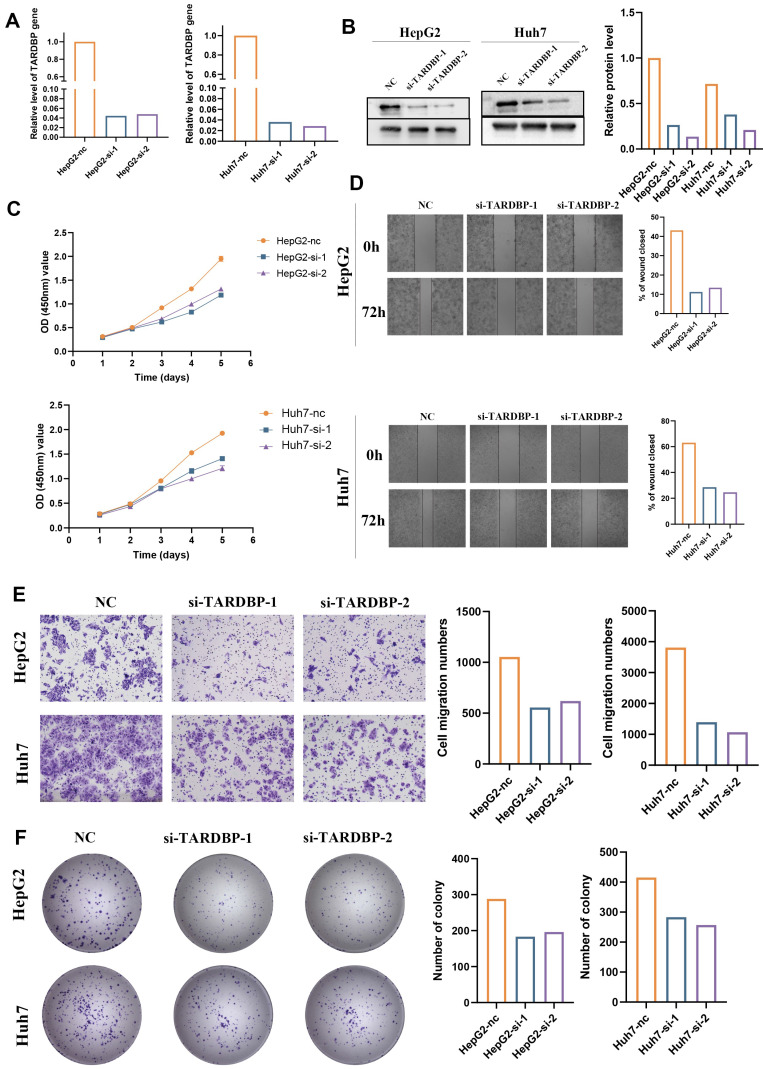
Functional Assessments of TARDBP Knockdown in HepG2 and Huh7 Hepatocellular Carcinoma Cell Lines. (A) Quantitative RT-PCR analysis displays a marked reduction in TARDBP mRNA levels in HepG2 and Huh7 cells transfected with two distinct siRNA sequences targeting TARDBP (si-TARDBP-1, si-TARDBP-2), demonstrating effective gene silencing compared to non-transfected controls (NC). (B) Western blot analysis corroborates the knockdown at the protein level, with a significant decrease in TARDBP protein expression observed in siRNA-treated HepG2 and Huh7 cells. Densitometric analysis quantifies the relative protein levels, further confirming the knockdown efficacy. (C) Cell proliferation assays, as indicated by OD measurements, show a decrease in the proliferative capacity of both HepG2 and Huh7 cells post-siRNA transfection, suggestive of TARDBP's role in cell growth. (D) Wound healing assays reveal impaired migration in both cell lines with siRNA-mediated TARDBP knockdown, indicated by the reduced wound closure percentage after 72 hours compared to the control group. (E) Transwell migration assays demonstrate that the siRNA-induced downregulation of TARDBP significantly hinders the migratory abilities of HepG2 and Huh7 cells, as reflected by the reduced number of cells migrating through the membrane. (F) Colony formation assays indicate a decrease in clonogenic potential in both HepG2 and Huh7 cells following TARDBP knockdown, with a noticeable reduction in the number of colonies formed by cells with suppressed TARDBP expression.

To delineate the functional role of TARDBP in liver cancer, HepG2 and Huh7 cell lines underwent siRNA-mediated TARDBP knockdown. Validation of knockdown efficacy was achieved through RT-qPCR, which confirmed a significant reduction in TARDBP mRNA levels in cells treated with siRNA-TARDBP-1 and -2 compared to untreated controls (P < 0.05; Figure 8A). Western blot analysis corroborated these findings, demonstrating a pronounced decrease in TARDBP protein expression following siRNA treatment (Figure 8B).

### 3.8. Functional consequences of TARDBP silencing on hepatocellular carcinoma cell proliferation, migration, and invasion

Subsequent analysis focused on the repercussions of TARDBP silencing on critical oncogenic behaviors in HepG2 and Huh7 cell lines. Proliferation was quantitatively assessed using CCK-8 assays, revealing a significant decline in cell growth post-siRNA-TARDBP-1 and -2 administration, a difference observable from 48 hours and persisting up to 120 hours post-transfection (P < 0.05; Figure 8C). The capacity for wound closure, a surrogate for cellular migration, was significantly impaired in TARDBP-silenced cells, as demonstrated by wound healing assays at 72 hours post-transfection (P < 0.05; Figure 8D). Additionally, transwell assays provided quantitative evidence of reduced migration and invasion abilities in these cells following TARDBP knockdown (P < 0.05; Figure 8E), and colony formation assays indicated a significant reduction in clonogenic potential under conditions of TARDBP depletion (P < 0.05; Figure 8F).

## 4. Discussion

The domain of cancer research, particularly within the study of hepatocellular carcinoma (HCC), has advanced dramatically, unveiling the intricate mechanisms driving oncogenesis and identifying potential therapeutic targets 16, 17, 21-23. Amidst these developments, TARDBP, traditionally linked to neurodegenerative disorders, has emerged as a molecule of interest within oncology, especially for its aberrant expression in HCC and other cancers 1, 24. TRecent studies reveal TARDBP pivotal role in malignant tumor progression. In breast cancer, it modulates the splicing of PAPR3 and NUMB mRNA via interaction with splicing factor 3, promoting tumor proliferation and metastasis 25. In lung cancer, TARDBP enhances FasL mRNA stability, inducing apoptosis and impeding tumor progression. Aberrant metabolic reprogramming, notably in glucose and lipid metabolism, is a hallmark feature of HCC, facilitating energy production and apoptotic evasion 26. In HCC, TARDBP upregulates the platelet isoform of phosphofructokinase, a pivotal glycolytic enzyme, promoting cellular proliferation 27, 28. Moreover, TARDBP, functioning as an RNA-binding protein, modulates ABHD2 RNA stability, thereby influencing fatty acid release and ROS levels, pivotal in regulating apoptosis and fostering HCC progression 29. Investigations into TARDBP's role in cancer have illuminated its contributions to critical cellular processes such as proliferation, apoptosis, and metastasis, suggesting its pivotal role in the malignant transformation and progression of HCC 30, 31. The exploration of TARDBP in oncological studies, particularly within the context of HCC, has shown promising results, with its aberrant expression correlating with adverse patient outcomes and tumor aggressiveness. These findings underscore the potential of TARDBP as a novel therapeutic target, offering new avenues for the development of targeted therapies that could significantly alter the treatment landscape for HCC and potentially other malignancies 32-36. The emerging research on TARDBP not only enriches our understanding of the molecular pathology of cancer but also paves the way for innovative approaches to cancer treatment, emphasizing the importance of molecular studies in identifying new targets for therapeutic intervention.

Our investigation into TARDBP's pan-cancer expression revealed its significant upregulation across a diverse array of tumor types, suggesting a universal role in cancer biology. This upregulation, particularly pronounced in specific cancers like THYM and TGCT, indicates its potential involvement in critical oncogenic processes. Furthermore, the positive correlation between TARDBP expression and TMB in several cancers underscores its potential impact on genomic instability, a hallmark of cancer progression. These findings are instrumental in understanding TARDBP's role in tumorigenesis and its implications for cancer treatment strategies.

The comprehensive analysis of immune and inflammatory-related factors in relation to TARDBP expression sheds light on its complex interaction within the tumor microenvironment. The positive association with immuno-stimulatory factors and an inverse relationship with immune inhibitors underscore TARDBP's potential influence on immune evasion mechanisms. Additionally, the negative correlation between TARDBP expression and both the StromalScore and ImmuneScore across many tumors further implicates its role in modulating the tumor microenvironment, potentially affecting response to immunotherapy.

The clinical implications of TARDBP's aberrant expression, particularly in HCC, where it is significantly associated with poor survival and higher WHO stages, highlight its potential as a prognostic marker. Moreover, the differential gene expression analysis in HCC patients based on TARDBP expression levels, followed by GO and GSEA analyses, suggests TARDBP's involvement in mRNA processing, small molecule catabolism, and lipid transport pathways. These pathways are crucial for cancer cell metabolism and may offer novel therapeutic targets. The strong predictive value of TARDBP expression for HCC incidence, as evidenced by ROC analysis, further supports its utility in cancer diagnostics.

Finally, the positive correlation of TARDBP with critical immune checkpoint molecules CD274 and CTLA4 opens new avenues for exploring its role in immune regulation within tumors. Immunotherapeutic interventions, which target the intricate milieu of the tumor microenvironment while modulating immune homeostasis, have exhibited promising efficacy across various solid tumors such as lung cancer, renal cancer, and melanoma 37, 38. Their mechanism of action primarily involves the inhibition of CD274 and CTLA-4 checkpoints. Presently, ongoing clinical trials are evaluating the therapeutic potential of checkpoint inhibitors in HCC 39.

Notably, data presented from the phase I/II CheckMate-040 trial at the 2019 annual meeting of the American Society of Clinical Oncology (ASCO 2019) revealed encouraging outcomes. Specifically, the combination therapy comprising Opdivo (PD-1 antibody) and Yervoy (CTLA-4 antibody) demonstrated an objective response rate of 31% with a notable response duration extending up to 17.5 months 40. These findings underscore the burgeoning significance and substantial therapeutic impact of immunotherapy in the management of HCC. This association might provide insights into designing combined therapeutic strategies targeting both TARDBP and checkpoint pathways to enhance anti-tumor immunity.

In summary, our findings position TARDBP as a potential new candidate for cancer therapy across multiple tumor types, especially HCC. Its broad impact on tumor biology, from genetic instability and immune regulation to influencing key metabolic pathways, underscores its significance as a therapeutic target. Further studies are warranted to explore TARDBP-targeted therapies and their integration into existing cancer treatment modalities, aiming to improve patient outcomes through precision medicine.

Finally, our thorough experimental investigations, including CCK8, colony formation, and Transwell assays, conclusively demonstrate that TARDBP significantly promotes the proliferation, migration, and invasion of hepatocellular carcinoma cells. These findings elucidate the crucial role of TARDBP in HCC progression and suggest its potential as a therapeutic target for HCC treatment.

There are several limitations to this study that need to be acknowledged. Firstly, the data utilized in this article were derived exclusively from the TCGA database alone, without inclusion from our center or external validation in other public databases or centers. Secondly, further explorations in vivo and in vitro are essential to comprehensively understanding the role of TARDBP in tumors, especially HCC. And our conclusions also need further animal experiments and subsequent clinical data to solidify our findings. In summary, through a comprehensive pan-cancer analysis, our study provides strong evidence for the prognostic and immunological value of TARDBP in various tumors, especially in HCC, which highlights the multifaceted roles of TARDBP and the potential clinical value it serves as a future target in liver cancer treatment.

## 5. Conclusion

In summary, our comprehensive pan-cancer analysis establishes robust evidence regarding the prognostic and immunological significance of TARDBP, with particular emphasis on its relevance to LIHC. These findings underscore the diverse functions of TARDBP and its promising clinical implications as a prospective target for liver cancer therapy.

## Supplementary Material

Supplementary tables.
