# Supplementary material for: TARDBP is a candidate diagnostic biomarker promoting tumor progression via impacting tumor immunity and tumor microenvironment
Source: J Cancer. 2024 Jun 3;15(13):4113–27. doi: 10.7150/jca.96800 (PMC11212099; doi:10.7150/jca.96800)
Supplement: Supplementary file 1 — Supplementary tables. [file jcav15p4113s1.zip › supplementary Table S2.docx]

Supplementary Table S2. Two TARDBP siRNAs sequence.

| TARDBP-siRNA1 | ACAACAUACACCAGAUUUCCCGAAAUCUGGUGUAUGUUGUCA |
| --- | --- |
| TARDBP-siRNA2 | UAAAUCGGAUGUUUUCUGGACCCAGAAAACAUCCGAUUUAAU |
