# Supplementary material for: TARDBP is a candidate diagnostic biomarker promoting tumor progression via impacting tumor immunity and tumor microenvironment
Source: J Cancer. 2024 Jun 3;15(13):4113–27. doi: 10.7150/jca.96800 (PMC11212099; doi:10.7150/jca.96800)
Supplement: Supplementary file 1 — Supplementary tables. [file jcav15p4113s1.zip › supplementary Table S1.docx]

Supplementary Table S1. Oligo sequences used in quantitative real-time PCR, inclduing Melting

temperature (Tm) and Annealing temperature (Tm-5℃).

| Target | Primer | Melting  temperature  (Tm) | Annealing  temperature  (Tm-5℃) |
| --- | --- | --- | --- |
| GAPDH | Forward  GAACGGGAAGCTCACTGG | 63.0℃ | 58.0℃ |
|  | Reverse  GCCTGCTTCACCACCTTCT | 65.3℃ | 60.3℃ |
| TADBP | Forward GGGTAACCGAAGATGAGAACG | 60.2℃ | 55.2℃ |
|  | Reverse CTGGGCTGTAACCGTGGAG | 62.0℃ | 58.0℃ |
